# Renessans Helps in Early Clearance of SARS-CoV-2: *In-Vivo* Activity of the Iodine Complex in *Rhesus macaque*

**DOI:** 10.3390/life12091424

**Published:** 2022-09-13

**Authors:** Muhammad Nawaz, Muhammad Adnan Ashraf, Muhammad Asad Ali, Muhammad Zubair Shabbir, Muhammad Abu Bakr Shabbir, Imran Altaf, Sohail Raza, Saira Rafique, Sohail Hassan, Nageen Sardar, Adnan Mehmood, Muhammad Waqar Aziz, Sehar Fazal, Muhammad Tahir Khan, Hafiz Muhammad Moavia Atique, Ali Asif, Zia Ullah, Mubashir Iqbal, Talha Imtiaz, Muhammad Anwar, Nadia Mukhtar, Tahir Yaqub

**Affiliations:** 1 Institute of Microbiology, University of Veterinary and Animal Sciences, Lahore 54000, Pakistan; 2 Pet Centre, University of Veterinary and Animal Sciences, Lahore 54000, Pakistan

**Keywords:** iodine compound, antiviral, COVID-19, coronavirus, animal trial

## Abstract

**Simple Summary:**

Coronavirus has caused a wide range of mortality all over the world. The disease is being controlled by vaccines and antiviral agents. Nevertheless, the development of emerging variants keeps on spreading the disease. For the control of the disease, researchers are always finding new alternatives for the treatment of COVID-19. We explored the potential of repurposing iodine complexes for the treatment of COVID-19. In the first stage, we tested the iodine complexes for antiviral activity in the lab and found the atoxic anti-coronavirus doses to be effective. In the current study, we tested the complexes in animal trials on monkeys. The iodine complexes helped to clear coronavirus from monkeys earlier than the control group. This study provides a cheap alternative to be tested for human trials for COVID-19 that can be a good additive option for treatment.

**Abstract:**

Iodine complexes have known antimicrobial properties along with reported *in-vitro* antiviral activity for several viruses. Renessans is one such product with iodine complexes and ascorbic acid. The present study was designed to determine its efficacy for SARS-CoV-2 in *Rhesus macaque*. *Rhesus macaque* were assigned to: A) prophylactic group (n = 3), (B) treatment group (n = 3), (C) infection control group (n = 4), and (D) negative control group (n = 4). Groups A, B, and C were challenged with 2 × 10^6^ TCID of SARS-CoV-2. The prophylactic group (A) was administered Renessans from 5 days before infection till 8 days postinfection (DPI). The treatment group (B) was administered Renessans from 3 till 8 DPI. Group C was administered water-insoluble fractions only. Nasal swabs from all monkeys of groups A, B, and C remained positive for SARS-CoV-2 till 2 and 7 DPI, while the swabs became negative for groups A and B at 14 DPI. Likewise, fecal matter of monkeys in group A returned negative results during the experiment, while that of group B had significantly decreased viral load (10^1.5^ genome copies/mL) compared to group C (10^3^ genome copies/mL). Hence, it is concluded that Renessans has *in-vivo* SARS-CoV-2 activity and may result in early clearance of SARS-CoV-2.

## 1. Introduction

Severe acute respiratory syndrome-related coronavirus-2 (SARS-CoV-2) was first reported as the etiologic agent of coronavirus disease 2019 (COVID-19) in December 2019 at a wholesale seafood market in Wuhan, Hubei, China [1,2]. According to the World Health Organization (WHO), more than 613 million confirmed cases and more than 6.5 million fatalities have been reported worldwide since its first report [3].

SARS-CoV-2 belongs to the Coronaviridae family and *Nidovirales* order. SARS-CoV-2 is a single-stranded unsegmented positive-sense RNA with a size of 30 kb. The virus can spread from one human to another through coughing and sneezing. The predilection site of the virus is lung alveolar epithelial type 2 (AT2) cells. Several studies have reported that the spike proteins of SARS-CoV-2 bind to angiotensin-converting enzyme-2 (ACE-2) receptors present on AT2 cells [4,5]. It has been reported that ACE-2 receptors are also present in the tubular epithelia of the kidney, pancreas, heart, and endothelial cells [4,6,7]. Upon entering the host cell, the virus releases its positive-sense RNA, which dictates host-cell machinery and produces new virions [8]. SARS-CoV-2 infection can be asymptomatic, and in most cases may cause mild to severe complications [9]. Given the prevalence of asymptomatic individuals and the limited availability of molecular testing in different parts of the world, it is believed that the correct number of infections may be much higher than the estimates [10].

Notably, many variants of concern and variants of interest of the virus have been developed, which has kept on challenging the ongoing prevention and therapeutic strategies [11,12,13]. There is a constant need for improvement in the vaccines and repurposing of the already available antiviral compounds. Researchers are continuously working on providing alternative treatment options for COVID-19. Lopinavir–ritonavir, chloroquine, favipiravir, and remdesivir have shown good potential [14]. Alternatively, repurposing of other compounds with known antimicrobial properties in the inactivation of enveloped and nonenveloped viruses has been explored [15]. Antiviral activity of iodine formulations has been reported for herpes simplex virus [16], coronaviruses [17], influenza virus [18,19], adenoviral conjunctivitis [20], hepatitis C virus [21], and African swine fever virus [22]. During the pandemic of COVID-19, iodine formulations were repurposed for their antiviral potential as nasal sprays, mouthwash, eyewash, antiseptics, and overall anti-SARS-CoV-2 potential [23,24,25,26]. A pharmaceutical product of an iodine complex by the name of Renessans is already being used to treat polycystic fibrosis [27]. The antibacterial, antiulcer, and immunomodulatory effects have already been documented [28]. The closest formulation is of Balsam “Vozrozhdenie.” It is an organic product with relatively less antimicrobial activity manufactured by MTI Medical based in Kazakhstan. The product is certified and is available in Kazakhstan, Kyrgyzstan, Russia, Belarus, Ukraine, and the UAE. The Renessans formulation has been approved by the Drug Regulatory Authority of Pakistan (DRAP registration 505620098). Renessans contains iodine (0.4–2.0%), potassium iodide (0.8–4.0%), starch (10.0–40.0%), ascorbic acid (0.4–2.0%), glucose (1.2–4.8%), and sodium chloride (0.3–1.8%) in weight. Our research group has previously reported the *in-vitro* antiviral potential of Renessans for SARS-CoV-2 [29]. Keeping in view the background, the present study was designed to further explore this alternative treatment for SARS-CoV-2 using monkeys (*Rhesus macaque*) as animal models.

## 2. Materials and Methods

### 2.1. Biosafety and Ethics Statement

High ethical standards were maintained during the conducting of experiments. Approval of the study was undertaken in compliance with the institutional guidelines of the ethics review committee (reference DR/317/7-7-202) of the University of Veterinary and Animal Sciences (UVAS), Lahore, Pakistan. Monkeys were housed in individual cages in a controlled comfortable environment in Animal Biosafety Laboratory-3 (ABSL-3) of the Institute of Microbiology (IM), UVAS. SARS-CoV-2-infected animals were housed under standard conditions of ambient temperature (22 ± 2 °C). Diagnostics of animal samples for the virus was performed in ABSL-3 for emerging pathogens (accreditation PHC/L&A/Lic/2020/COVID-13). A cycle of 12 h light and darkness was maintained throughout the experiment. Food comprising fruit and bread loaves was provided twice daily by trained staff and water provided to monkeys ad libitum throughout the experiment. Animals were kept under the supervision of a veterinarian.

### 2.2. Experimental Design

The monkeys were obtained from the wildlife department of Pakistan to determine the *in-vivo* efficacy of the antiviral drug (Renessans) for SARS-CoV-2. Before housekeeping, animals were tested for any communicable disease. Healthy ones were weighed and divided into four groups: (A) prophylactic (n = 3), (B) treatment group (n = 3), (C) infection control (n = 4), and (D) negative control (n = 4). SARS-CoV-2 (GenBank accession number MW031802) infection @ 2 × 10^6^ TCID was given to groups A, B, and C through intranasal and oral routes (0.5 mL each) under anesthesia (mixture of ketamine and xylazine). Toxic (50 µg concentration) and effective (EC50 = 0.425 µg/mL) doses of the antiviral drug were calculated in our previous *in-vitro* study (Altaf et al., 2021). The antiviral drug (Renessans) was administered intravenously (IV) at 2.85 mg/7 kg (once daily) to group A from 5 days before the infection till 8 days postinfection (DPI). Group B was administered Renessans intravenously after the onset of clinical signs and symptoms from 3 to 8 DPI at 2.85 mg/7 kg (once daily). Groups C and D were administered IV with water-insoluble fractions only.

### 2.3. Fecal and Nasal Swab Sampling

**Figure 1:**
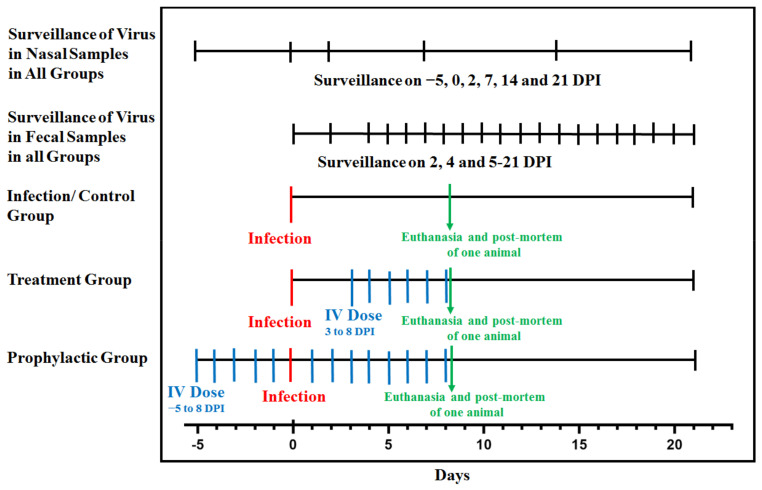
Detailed timeline of events for determining the *in-vivo* efficacy of the antiviral drug (Renessans) for SARS-CoV-2.

Fecal and nasal swab sampling was performed to determine the shedding of SARS-CoV-2 through these routes. All monkeys of groups A, B, C and D were anesthetized for nasal sampling. Nasal sampling was performed five times during the whole experiment: firstly at 0 day before the infection and then at 2 DPI, 7 DPI, 14 DPI, and 21 DPI from all monkeys of groups A, B, and C. Fecal samples were collected daily from day 0 to 21 DPI. The experimental design for A, B and C groups is given in Figure 1.

### 2.4. RNA Extraction from Fecal and Nasal Swab Samples

Fecal and swab samples were subjected to RNA extraction in ABSL-3. For RNA extraction, each sample was vortexed, 1 mL transferred to a microcentrifuge tube followed by centrifugation at 5000 rpm for 10 min at 4 °C, and supernatant was collected in a new microcentrifuge tube. The Hero-32 extraction system (Luoyang Ascend Biotechnology Co., Ltd., Luoyang, China) was used for RNA extraction. A total of 14 µL of proteinase K+ carrier RNA mixture was added to each well of the RNA extraction plate, followed by the addition of a supernatant (fecal and nasal swab samples) of 200 µL into the respective wells. The extraction plate was placed in an RNA extractor and RNA was extracted from the elution wells of plates and stored at −80 °C. RT-qPCR analysis was performed on the same day.

### 2.5. Detection of SARS-CoV-2 from Fecal and Nasal Swab Samples by Real-Time Quantitative PCR

A one-step RT-qPCR kit (Maccura Biotechnology, China) was used to synthesize the cDNA. The CFX96 touch real-time PCR (Bio-Rad) was used for amplification of the *ORF1ab* gene. The cyclic conditions were preincubation at 95 °C for 2 min, followed by 40 cycles of 15 s at 95 °C and 40 s at 95 °C. MS2 based pseudovirus containing exogenous RNA sequence serving as an internal process control (Maccura Biotechnology, Chengdu, China) was used as an internal codon for normalization. Reactions with a cycle threshold below 40 cycles were assumed positive. A standard curve was developed for the CT values of known concentration of log_10_ copies/mL of SARS-CoV-2. Genome copies of SARS-CoV-2 were calculated with the help of a standard curve [30].

### 2.6. SARS-CoV-2 Detection from Tissue by Real-Time Quantitative PCR

Three monkeys (one each from groups A, B and C) were euthanized for the determination of SARS-CoV-2 from different tissue samples by RT-qPCR. These monkeys were euthanized by intracardiac injection of potassium chloride 10 mL. Intestine, heart, lung, trachea, and ovary tissue samples were excised and stored at −80 °C till further use.

## 3. Results

### 3.1. SARS-CoV-2 Detection from Fecal Samples of Monkeys by Real-Time Quantitative PCR

**Figure 2:**
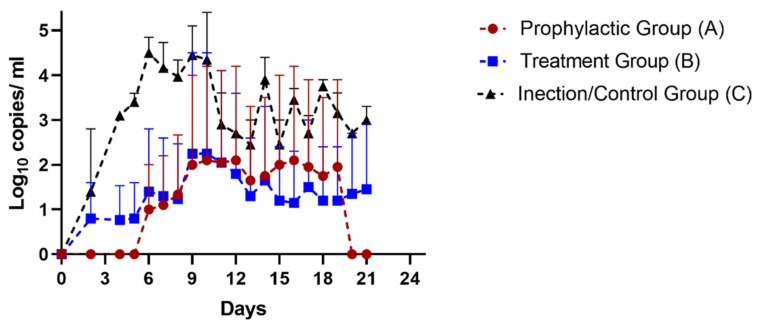
Longitudinal comparison of average fecal viral load of experimental and control groups.

Fecal samples were collected from A, B, C and D group monkeys at different times (Figure 1) for the detection of SARS-CoV-2 by real-time qualitative RT PCR. At 4 DPI and onwards, all three monkeys remained positive in C group while the virus was detected from fecal matter of one monkey of group A (P2) and B (T3). All the monkeys of D group remained negative. Noteworthily, at 21 DPI, P2 monkey from A group was found negative for SARS-CoV-2 and a group B (T3) monkey remained positive for SARS-CoV-2 (Supplementary Table S1). All monkeys of group C were still shedding the virus. Fecal matter of monkeys in the prophylactic group returned negative results at 20 DPI, while the monkeys of the treatment group revealed an average of less viral load (10^1.5^ copies/mL) than the infection control group (10^3^ copies/mL) (Figure 2, Supplementary Table S1). These findings suggested that Renessans has *in-vivo* SARS-CoV-2 activity and may result in early clearance of SARS-CoV-2.

### 3.2. SARS-CoV-2 Detection from Nasal Swab Samples of Monkeys by Real-Time Quantitative PCR

**Figure 3:**
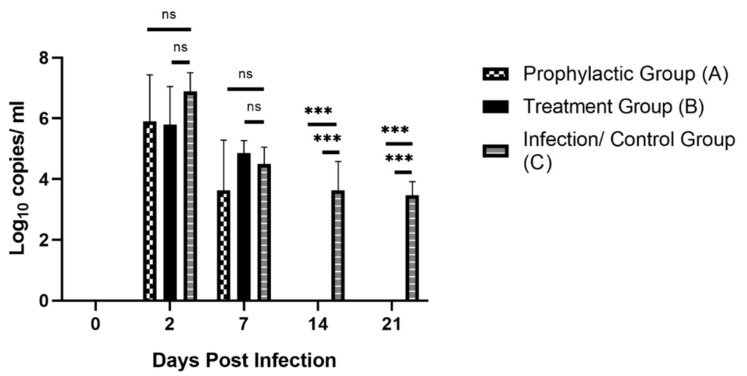
Graphical representation of the comparison of average nasal viral load of experimental and control groups. ***: *p* < 0.01, ns: non-significant.

Nasal swabs were collected from A, B and C groups at different times for the detection of SARS-CoV-2 by real-time qualitative RT PCR. Preinfection nasal swab sampling was also performed to detect SARS-CoV-2 by real-time quantitative PCR from all the monkeys of groups A, B and C to rule out any previous exposure to SARS-CoV-2. However, after 48 h of infection, all the monkeys from groups A, B and C were found positive for SARS-CoV-2. At 2 and 7 DPI, there was no significant difference in the viral load of prophylactic and treatment group compared to group C. However, all (100%) nasal swabs from prophylactic and treatment groups were negative for SARS-CoV-2 at 14 and 21 DPI. Nevertheless, monkeys of the infection control (C) group were still found positive for SARS-CoV-2, and the viral load remained significantly high compared to the experimental groups (negative) at 14 (an average of 10^3.6^ copies/mL) and 21 (an average of 10^3.4^ copies/mL) DPI (Figure 3, Supplementary Table S2). All the monkeys of group D remained obviously negative in the negative-control group, therefore, results are not displayed in figures and tables. Based on these findings, it can be reported that Renessans (antiviral drug) helped in the early recovery of group A and B monkeys from SARS-CoV-2.

### 3.3. SARS-CoV-2 Detection from Tissue of Group A, B and C Monkeys by Real-Time Quantitative PCR

For determining SARS-CoV-2 replication in body tissue, one monkey was euthanized at 2 DPI for postmortem from group C to confirm the infectivity potential. The animal was positive for SARS-CoV-2 in its lungs, trachea, and heart. Later, three monkeys (one from each of groups A, B and C) were euthanized at 8 DPI. SARS-CoV-2 detection from different tissue types was performed by real-time quantitative PCR. SARS-CoV-2 was detected in its intestine, lung, heart, ovary, and trachea tissue. At 8 DPI, the tissue of monkeys in the infection control group (group C) were positive for SARS-CoV-2, while the genome was not detected in groups A and B (Table 1). These findings also suggested that the antiviral drug Renessans protected against systemic SARS-CoV-2 infection.

## 4. Discussion

Morbidity and mortality due to COVID-19 are constantly on the rise, as many variants of SARS-CoV-2 have developed. Although different COVID-19 vaccines have reduced the transmission of the disease along with some of the antivirals, nevertheless, there is a need to explore more alternative therapeutics. Therefore, the already tested *invitro* potential of an iodine complex [29] was subjected to preclinical studies in the present study. Renessans, the iodine complex, proved its potential *in-vivo* by leading to early clearance in monkeys when used as prophylaxis and treatment compared to the control group. Therefore, iodine complexes can be further investigated and used in animal and human trials on a larger scale. Iodine compounds have been previously reported to bring about changes in the viral coat by modifying histidine and tyrosine amino acids leading to impairment of the attachment phase [31]. The present findings are consistent with the studies performed on MERS coronavirus and SARS-CoV-2 where researchers found iodine compounds to be clearing the viral load in cell-culture suspension assays, oral cavity, skin surface, and nasopharyngeal wash [23,24,32,33,34]. The present study’s findings may pave the way for the repurposing of Renessans, which is already approved for the treatment of ulcerative conditions (European Patent Specification 2011).

Iodine compounds are known for their antimicrobial activity for topical treatment; however, different studies are exploring the possibilities of their use as oral and parenteral formulations. Systemic use can be associated with toxicity. Therefore, there was a need for evaluation of the toxicity potential of Renessans. The toxicity dose of Renessans was determined by MTT assay on Vero cell lines in the preliminary *in-vitro* study [29]. Researchers had reported the antiviral activity of iodine compounds for the family of coronaviruses even before the spread of SARS-CoV-2 [17]. One of the most studied compounds was povidone–iodine, which revealed its potential on cell lines *in-vitro* and on clinical trials *in-vivo*. Povidone–iodine formulations of antiseptic solution, skin cleanser, gargle, and mouthwash were effective in killing 99.9% of viral load when tested via suspension assays on Vero E6 cell lines [34], and cleared the viral presence *in-vivo* when used as a mouthwash in the oral cavity of four patients [23]. A concentration containing 1% active ingredient showed complete virucidal effect on salivary secretions [24]. The results of virucidal activity remained promising when the formulation was tested via nasal irrigation for nasopharyngeal wash as well [33]. Apart from other formulations of iodine, Renessans composition has also been tested for antiviral potential for influenza virus and hepatitis C virus [21,35]. As Renessans had shown potential for antiviral activity, a gap existed in the evaluation of iodine complexes in animal models, which the present study fills.

Baboons, marmosets and ferrets have been used as animal models for COVID-19 studies. Transgenic mice with hACE receptors and nonhuman primates have shown good potential for studies on the progression of SARS-CoV-2 infection. However, *Rhesus macaque* were selected as a more suitable nonhuman primate animal model for the present study, as there is variability of expression of hACE across different organs in transgenic murine models [36]. SARS-CoV-2 might not replicate in extrapulmonary organs in murine models [37]. Murine models have the limitation of not showing high SARS-CoV-2 viral loads as well. Furthermore, *Rhesus macaque* ACE has the highest receptor activity for SARS-CoV-2 of 14 mammalian species [38], and positivity or clearance of infection can be determined based on shedding of virus from nasal secretion, fecal matter, and molecular detection from body tissue after biopsy [39]. SARS-CoV-2 is excreted in fecal matter, which is a reliable source of early detection compared to nasopharyngeal samples. The results of detection from fecal matter demonstrated that Renessans showed better antiviral potential when used as prophylactic than as treatment, as monkeys in the A group returned negative in 20 days, while the average viral load for monkeys of B group decreased to 10^1.5^ copies/mL compared to group C’s 10^3^ copies/mL. A likely reason for clearance is perfused availability of iodine to systemic organs in the animals of prophylactic groups, as their dosage was started 8 days before the treatment group. As one animal from each group was euthanized for the possible detection and quantification of viral load in body tissue, the sampling results after death are termed “not applicable” (NA) in Supplementary tables.

Nasopharyngeal and nasal samples are also good alternative options to track the high or low titer of SARS-CoV-2. However, only nasal sampling was feasible due to the conventional size of swab buds and the short nostril passage of the monkeys. Notably, the highest viral load in C group reached 10^6.9^ copies/mL, which later decreased to 10^3.4^ copies/mL at 21 DPI. However, the viral genome copies reached 10^5.8^ copies/mL in A and B groups and later became negative at 14 DPI in nasal swab samples. One of the unusual findings was that animals from all the experimental groups became positive for SARS-CoV-2 on 2 DPI after challenge infection via nasal sampling; however, two animals from T1 and T2 appeared negative till 2 DPI via fecal sampling (Supplementary Table S1). Presumptively, onset of shedding of the virus in fecal matter was delayed compared to nasal sampling; however, shedding of virus stopped earlier in nasal samples than in fecal matter.

After the completion of the 21-day preclinical study, we kept on testing the animals for the virus in fecal matter and nasal sampling on a weekly basis. Becoming negative for nasal swab sampling is suggestive of clearance of pulmonary organs and overall progression to clearance of infection. Duration of positivity of SARS-CoV-2 remains less when detected by nasal sampling; however, duration of positivity remains relatively higher for fecal matter [40]. The treatment B group became negative at 28 DPI, while the infection control C group became negative for nasal swab and fecal matter at 28 DPI and 35 DPI, respectively. Alleviation of shedding of the virus was an interesting finding, in contrast to the study of Williamson et al., in which SARS-CoV-2 kept on shedding after the use of remdesivir [41]. Presumably prophylactic (A) and treatment (B) groups recovered early because of SARS-CoV-2 specific immune response as well [42,43]. Nasopharyngeal swab sampling is a standard method to detect SARS-CoV-2 [44], and hence was utilized in the study. Based on these findings, it can be reported that Renessans did have a positive effect and helped in the early recovery of infected monkeys.

The replication of virus in lungs and extrapulmonary body tissue is an important criterion for the progression of systemic viral infection and an important aspect of the study as well. The *Rhesus macaque* is a suitable animal model that can demonstrate the systemic infection of SARS-CoV-2 by having the potential of replication of the virus in extrapulmonary organs [36], as the ACE-2 receptors present in type II pneumocytes, nasal goblet secretory cells, and absorptive enterocytes share more than 91% homology with human ACE-2. Therefore, the experiment was designed to determine the susceptibility of body tissue to SARS-CoV-2. Upon real-time quantitative PCR of postmortem tissue samples, the virus was tested from lungs, trachea, ovary, intestine, and heart. The viral load in different tissue types of C group ranged from 10^2^ to 10^5.4^ genome copies/mL. However, the virus was not detected on testing the tissue of A and B groups. Our time for viral detection in body tissue was justified as the viral load in lower respiratory tract becomes maximum at 9 DPI after intranasal inoculation [39]. The findings suggested that Renessans may have provided protection from systemic infection. Furthermore, SARS-CoV-2 can infect other organs apart from the lungs in C group. Similar findings were also observed in other studies where researchers detected SARS-CoV-2 in brain, lungs, trachea, spleen, kidney, eye, gastrointestinal tract, uterus, lymph nodes, heart and liver [45,46,47].

Considering the influence of iodine on functioning of thyroid glands, safety studies of Renessans have been completed in phase II clinical trials (license CT-0014, reference F 3-47/2020-DD (PS). One of the limitations of the study was that the disease progression and therapeutic response could not have been studied in severe or later stages of SARS-CoV-2 infection. Trials have already been initiated to test the efficacy of other iodine complexes [48]. It is recommended to extrapolate the trials to the next levels so clinicians can tackle the disease with multiple therapeutic options.

## 5. Conclusions

In light of the current study findings, it is concluded that Renessans has an *in-vivo* SARS-CoV-2 activity and may result in early clearance of SARS-CoV-2. Therefore, the current study may provide a basis for a clinical trial of the drug in SARS-CoV-2 patients and reveal its anti-SARS-CoV-2 potential.

## Figures and Tables

**Table 1:** Log_10_ SARS-CoV-2 genome copies/mL from tissue of group A, B and C monkeys by real-time quantitative PCR at 8 DPI.

	Prophylactic (A Group)	Treatment (B Group)	Infection (C Group)
Organs	P3	T2	I2
Intestine	0	0	3
Lungs	0	0	5.4
Heart	0	0	3
Ovary	0	NA	2
Trachea	0	0	2.7

NA: not applicable.

## Data Availability

Data are available in the manuscript.

## Supplementary Materials

The following supporting information can be downloaded at: https://zenodo.org/record/6973267#.Yw90Q31BzIV. The following supporting information is available: Table S1: Log_10_ SARS-CoV-2 genome copies/mL from fecal samples of experimental and control groups by real-time quantitative PCR; Table S2: Log_10_ SARS-CoV-2 genome copies/mL from nasal swab samples of experimental and control groups by real-time quantitative PCR.
